# Serum Levels of Neuropeptides in Cows with Left Abomasal Displacement

**DOI:** 10.3390/vetsci5040103

**Published:** 2018-12-17

**Authors:** Marlene Sickinger, Joachim Roth, Klaus Failing, Axel Wehrend

**Affiliations:** 1 Clinic for Obstetrics, Gynecology and Andrology of Large and Small Animals with Ambulatory Service, Faculty of Veterinary Medicine, Justus-Liebig-University, 35392 Giessen, Germany; Axel.Wehrend@vetmed.uni-giessen.de; 2 Institute of Veterinary Physiology, Faculty of Veterinary Medicine, Justus-Liebig-University, 35392 Giessen, Germany; Joachim.Roth@vetmed.uni-giessen.de; 3 Biomathematics and Data Processing Unit, Faculty of Veterinary Medicine, Justus-Liebig-University, 35392 Giessen, Germany; Klaus.Failing@vetmed.uni-giessen.de

**Keywords:** dairy cattle, left abomasal displacement, serum, substance P, VIP

## Abstract

Abomasal displacement (AD) to the left is a common disease in high-yielding dairy cows after parturition. In view of the previously reported changes in tissue neuropeptide concentrations in cows with AD, the primary aim of this study was to evaluate the effect of AD and breed on serum neuropeptide concentrations. For this purpose, blood samples of 33 German Holstein (GH) cows with AD, 36 healthy controls (GH), and 32 healthy German Fleckvieh (GF) cows were collected, and concentrations of substance P (SP), vasoactive intestinal polypeptide (VIP), and interleukin1β (IL-1β) were measured via commercially available ELISA kits. To examine the effect of AD, we compared GH cows with and without AD and observed no significant effects of AD on SP, VIP, or Il-1 β concentrations. To evaluate the effect of breed, we compared healthy GH with healthy GF cows and detected markedly higher VIP serum levels in the healthy GF cows (*p* < 0.01). No significant differences in SP or IL-1β were detected. According to our results, there seems to be no effect of AD on the serum concentrations of SP, VIP, or IL-1 β. In contrast, there seems to be a breed difference concerning serum VIP concentrations.

## 1. Introduction

A multitude of risk factors for the development of abomasal displacement (AD) in cows have been described, including early lactation, breed and genetics, nutrition and body condition, puerperal diseases, and electrolyte disturbances [1,2]. Atony of the abomasum and associated reduced abomasal emptying rates are regarded as prerequisites for the onset of abomasal displacement [3]. The reduced abomasal emptying is also seen after surgical correction of the displacement, and therapeutic efforts have been made to treat these postoperative paralytic states of ileus [4]. Preoperatively administered erythromycin has been shown to improve postoperative abomasal emptying rates [4]. The positive effects of the motilin receptor agonist erythromycin on the motility of the gastrointestinal tract indicate the important role of neuropeptides in the pathogenesis of AD. Correspondingly, different neuropeptide concentrations—including those of substance P (SP) and vasoactive intestinal polypeptide (VIP)—have been described within the abomasal walls in German Holstein (GH) cows and German Fleckvieh (GF) cows, in which AD only occurs rarely [5]. The hypotheses of the current study were that there may be differences between serum SP and VIP concentrations in cows suffering from left abomasal displacement compared to healthy controls and between serum neuropeptide concentrations in healthy GH cows and GF cows. As displacement of the abomasum is often accompanied by inflammatory processes [6], serum concentrations of the cytokine interleukin 1β (IL-1β), a marker of inflammation, were also examined.

## 2. Materials and Methods

### 2.1. Blood Samples

Blood samples were collected from 33 GH cows with AD, 36 healthy control GH cows, and 32 healthy GF cows, and neuropeptide concentrations of SP and VIP were measured via commercially available bovine-specific ELISA kits (USCN Cloud-Clone Corp., Houston, TX, USA). IL-1β levels were determined using another bovine-specific ELISA kit (USCN Cloud-Clone Corp., Houston, TX, USA), and the results in each group were compared. Quality control results are given in Table 1. Cows were classified as healthy if there were no clinically detectable signs of disease (i.e., rectal temperature < 39.0 °C, good general condition, no lameness, and no signs of inflammatory processes). Healthy controls came from five different farms, and cows with AD came from 24 different farms. Age ranged between 2.3 and 11.8 years in GF cows (4.9 ± 2.4), between 2.2 and 10.7 years in healthy GHs (5.4 ± 2.2), and between 2.1 and 6.9 years in GH cows with AD (4.4 ± 1.4). Mean parity for all groups was 3.0 ± 2.2 with a mean of 169.4 ± 145.5 days in milk (DIM). Blood samples were collected and allowed to clot overnight at 4 °C. Centrifugation was performed for 20 min at approximately 1000× *g*. Serum samples were stored at −80 °C prior to examination.

All blood samples were either harvested from cows in accordance with the standard operating procedures of the Clinic for Obstetrics, Gynecology, and Andrology of Large and Small Animals, University of Giessen, Germany, or from samples that had been sent to the laboratory for diagnostic purposes. Control blood samples were derived from cows that underwent bloodwork for the purposes of herd health management and diagnostics. Sample collection was performed within 1 year (2014–2015) after notifying the local ethics authority and conformed with national ethics guidelines and legislation (internal number of correspondence kTV 11-2018).

### 2.2. Statistical Analysis

To assess homogeneity of the groups, a Kruskal–Wallis one-way analysis of variance was performed. Concerning age and parity, there were no statistically significant differences between the groups (age: *p* = 0.2; parity: *p* = 0.9). The lactation state of the examined animals was statistically significant in difference with *p* = 0.0002. A correlation between days in milk and the neuropeptide serum concentrations could not be shown, however.

In both comparisons, the healthy GHs represent the control group for the intended pairwise comparisons. For the variables SP and VIP (log-transformed serum) one-way analysis of variance (ANOVA) with subsequent pairwise group comparison with the control group incorporating the multiple t-test with correction of the significance level using the Bonferroni–Holm [7] method was performed to assess differences between the healthy GH cows and those with AD on the one hand, and between the GH cows and the healthy GF cows, on the other hand (statistical program package BMDP, test version BMDP7D, Los Angeles, CA, USA) [8]. Because the statistical distribution of VIP was skewed to the right, its values were logarithmically transformed in the analysis. Therefore, for VIP, data description was done by means of the geometric mean and geometric standard deviation, also called dispersion factor. As a considerable proportion of the IL-1β values were below the detection limit, no normal distribution was found, and for the parameter IL-1β group, comparison was performed via the non-parametric exact Kruskal–Wallis test (Cytel Studio StatXact Vers. 9.0.0, Cambridge, MA, USA) [9].

The assessment of normality of the variables was done by visual adspectation of the histograms. Additionally, a residual analysis was done by the Q-Q-plot. Variance homogeneity was assessed by the Levene test and all applied tests were two-sided. The significance level of α = 0.05 was chosen.

## 3. Results

**Figure 1:**
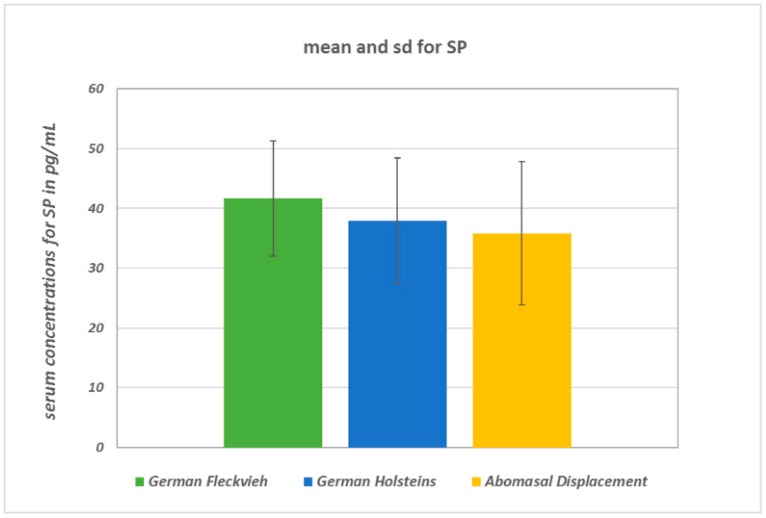
Mean concentrations and standard deviations of serum SP (substance P) concentrations. There were no significant differences between the groups. The comparison between healthy GF (German Fleckvieh) cows and the GH control cows resulted in *p* = 0.31 and in *p* = 0.43 between the GH (German Holstein) controls and the GHs with AD (abomasal displacement), respectively.

**Figure 2:**
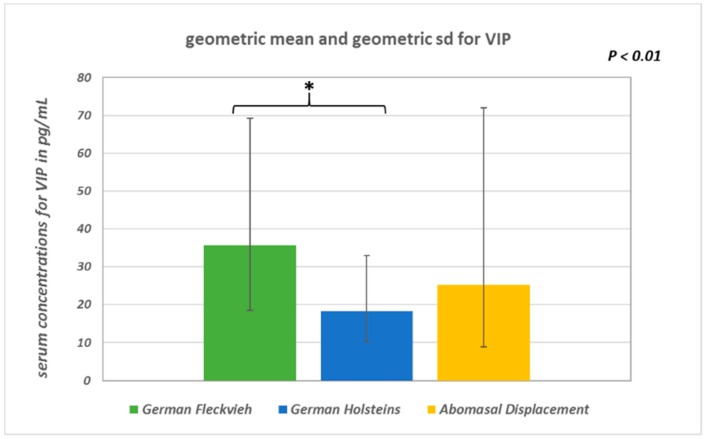
Geometric means and geometric standard deviations of log-transformed serum VIP (vasoactive intestinal polypeptide) concentrations (*p* < 0.01).

**Figure 3:**
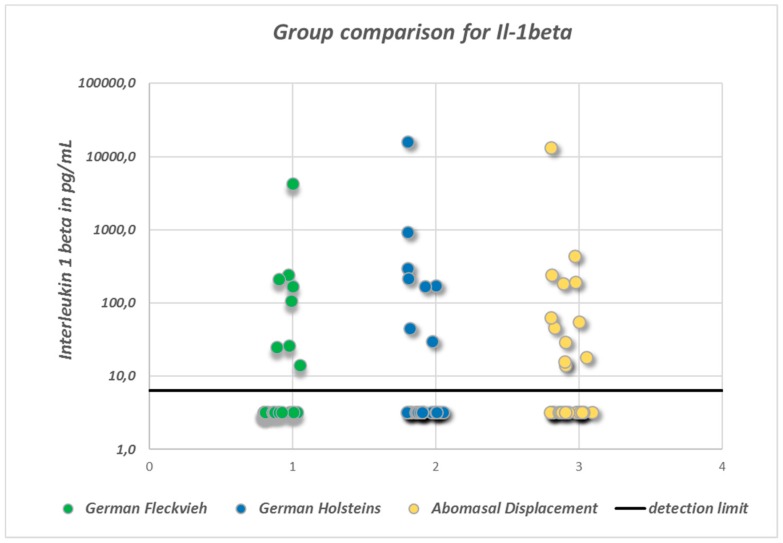
Scatter-plot indicating the distribution of serum interleukin1β (IL-1β) concentrations. Note the high proportion of values below the minimum detection limit of 6.4 pg/mL. In order to display these unknown concentrations, an arbitrary value of ½ of the minimum detection limit has been assigned to these samples. There were no statistically significant differences between the groups.

SP concentrations did not differ significantly between the groups (ANOVA: *p* = 0.094; Figure 1). In healthy GH cows, the mean SP concentration was 37.9 ± 10.5 pg/mL, whereas in GH cows with AD it was 35.8 ± 12 pg/mL, and in healthy GF cows it was 41.7 ± 9.7 pg/mL. There were statistically significant global differences in log-transformed VIP concentrations between the groups (ANOVA: *p* = 0.0033). Pairwise group comparison revealed a significant difference between GH cows (18.4 ∙ 1.8^±1^ pg/mL; geometric mean and geometric SD) and GF cows (35.7 ∙ 1.94^±1^ pg/mL) (multiple *t*-test with correction according to Bonferroni–Holm: *p* = 0.002; Figure 2). Serum VIP levels in GH cows with AD (25.2 ∙ 2.85^±1^ pg/mL) did not differ from those of the healthy controls (*p* = 0.1). No significant differences between the median IL-1β values of the three groups were detected (Figure 3). In all groups, the median value was lower than the detection limit.

## 4. Discussion

### 4.1. Effect of AD on Serum Neuropeptide Concentrations

In contrast to former reports of a significant reduction in neuropeptide concentrations within the abomasal walls of cows suffering from AD [10] and the expected higher serum concentrations in these cases, in the current study there were no significant differences in serum SP or VIP concentrations between healthy and diseased cows. This is dissimilar to results pertaining to other motility influencing substances such as ghrelin, motilin, and gastrin, of which enhanced serum concentrations have been described in cows with AD [11]. However, GH cows with AD exhibited a higher number of VPAC1/VIP-1 receptors than healthy controls, indicating a predominance of atonic neuropeptides in cows with AD [12].

In cases of AD, concomitant diseases such as ketosis, lameness, or metritis are very common [1]. Since these diseases were suspected to especially influence the concentration of SP [13], cows presenting these diseases were excluded from the presented study. Furthermore, there are evidently numerous local effects of SP including pain perception, induction of angiogenesis, and inflammation [14], that may also be present within the abomasal wall and may not be correlated with serum SP concentration. For instance, the involvement of SP in the opening processes of the cervix during calving is presumed because of an enhanced SP serum level in cows during physiological calving. In contrast, a torsion of the uterus resulted in no increase of SP when comparing those cows with healthy controls [15].

This multiplicity of in vivo effects of SP may be responsible for the lack of a significant difference between the cows with AD and the healthy controls in the current study. When examining SP as a mediator of pain or an indicator of stress, for example [16,17], there are reportedly enhanced serum concentrations in calves undergoing castration or physical restraint. With regard to our results, abomasal displacement thus may not be a condition that is accompanied by severe pain or stress. This is concordant with the clinical presentation of only mild if any colic symptoms and vital parameters being within the reference ranges in cows with left AD [11]. Different observations would be likely made in cows with abomasal volvulus.

The cytokine IL-1β is a marker of inflammation and has been used as a control parameter to indicate possible influencing effects of hidden inflammatory conditions on the concentrations of the neuropeptides SP and VIP in the current study. In the previously published literature, however, IL-1β concentrations have been reported to range from 33.1 to 95.1 pg/mL in cows after calving and physiological puerperium [18] up to concentrations of 500 pg/mL and 400,000 pg/mL in clinically healthy cows. Compared to this clearly huge variation in IL-1β concentrations in clinically healthy (or at least apparently clinically healthy) cows, the maximum detected IL-1β concentration of 15,932 pg/mL in the current study is presumed to be correlated to rather mild if any inflammatory processes. As there was also a substantial number of samples that evidently contained IL-1β concentrations that were below the detection limit (6.4 pg/mL), the results of the study indicate that there were only single cows suffering from inflammatory processes at the time of sampling. Severe inflammation therefore seems not to be a characteristic element of abomasal displacement to the left.

### 4.2. Effect of Breed on Serum Neuropeptide Concentrations

Unlike the SP concentrations, there was a significantly higher concentration of serum VIP in GF cows than in GH cows. Comparing this result to the previously published tissue neuropeptide concentrations in GF cows, it may be that there is a relationship between a smaller amount of VIP within the abomasal wall and an elevated serum VIP concentration. Assuming that the production of VIP within the nerve vesicles is equal in the two breeds compared in the present study, there may be a differential rate of transport out of the tissue and into the blood in the two breeds. Another possible explanation is that there is a reduced number of VIP receptors in GF cows; however, an immunohistochemical study investigating this detected no significant differences between GF and GH cows in this respect [12].

As presented above, the two groups of healthy GF cows and healthy GH cows did not differ concerning the inflammation marker IL-1β, indicating that there were no undetected concomitant diseases that could have influenced the results of the study. Therefore, breed seems not to have an effect on the susceptibility to inflammation.

### 4.3. Study Limitations and Future Attempts to Improve Study Outcome

There are several possible reasons for the failure to detect the expected enhanced serum neuropeptide concentrations in the current study. Because SP is a neuropeptide, it is prone to enzymatic degradation. Sample handling and preparation procedures may have an influence on the concentration of neuropeptide within a sample. The necessity of processing samples that are intended to be used for the analysis of SP concentrations in plasma samples within 1 hour of collection has been emphasized [19]. If an immediate analysis is not possible, chilling the samples or using enzyme inhibitors is suggested. According to another study, tachykinin degradation begins as early as 10 min after blood sampling [20]. Due to technical reasons, blood samples that were analyzed in the current study could not be processed and analyzed immediately, but sample preparation and serum harvesting were performed with outstanding accuracy and precise adherence to the manufacturer’s instructions. Nevertheless, neuropeptide degradation may have occurred to some extent, and it may have influenced the blood concentrations. Additionally, due to technical reasons no peptide extraction step was included in the current study. Therefore, we cannot exclude cross-reactivity bias, although the manufacturer of the SP-ELISA kit asserts that there is no significant cross-reactivity and guarantees a minimum detectable dose of SP of less than 4.64 pg/mL, which is three times less than the minimum concentration measured in our study.

In contrast to the above cited assertion that a comparison of SP concentrations between groups should only be performed if neuropeptide degradation is precluded [19], we believe that such comparisons may be valid if all samples are handled identically and the comparisons are restricted to samples analyzed within the same study. Problems of interpretation may arise if inter-study comparisons are performed. However, due to the lack of reliable normative SP values, the results of the present study should be interpreted critically, regardless. For example, normative SP values in human neonates reportedly range between 1.32 pg/mL and 15.09 pg/mL after solid state extraction and enzyme inhibition. Another study using neither extraction nor enzyme blocking yielded a reference range of 27.2–38.3 pg/mL [20]. Both normative values are < 240 pg/mL, which is stated as a reference value for SP in human adults by the American Medical Association [21]. In calves, mean SP concentrations of 452.7 ± 92.1 pg/mL have been reported using solid-phase extraction but no enzyme blocking [16]. For healthy ruminant cattle, plasma values of 250 pg/mL ± 90 pg/mL are given in one study [13], but no reference ranges for serum levels of SP are available in the literature. Notwithstanding some potential technical concerns, the results of the current study may serve as a first attempt to define normative values in adult cattle. According to these results, a normative value in the serum of cattle could be presumed to be < 55 pg/mL. However, there may be a discrepancy between serum neuropeptide concentrations in the peripheral blood and the concentrations of these neuropeptides within the local blood vessels supplying the abomasum. Although scientifically interesting, such an invasive sampling technique could not be performed within the present study because of animal welfare reasons.

The failure to detect statistically significant differences in SP concentrations in the groups in the current study may have been due to a considerable inter-individual variation with regard to the concentration of this neuropeptide. Furthermore, the AD group consisted of cows that had calved about 50 days before the occurrence of AD, in contrast to the control group of cows that were approximately 200 days in milk. This difference in the state of lactation might have led to a discrepancy within the results, although no statistically significant correlation could be found between DIM and any of the examined neuropeptide concentrations. Nevertheless, a matching of days in milk may be reasonable for future studies.

## 5. Conclusions

In GF cows, the previously reported higher baseline tonus associated with abomasal motility may be related to a higher serum concentration of VIP. The fact that in this breed’s AD to the left only occurs rarely may be relevant in this regard. In GH cows, there is evidently high inter-individual variation in serum neuropeptide concentrations, which may be associated with their known variation in predisposition for AD. Furthermore, there is evidently a lack of normative serum neuropeptide concentrations in cattle available in the literature. Therefore, studies conducted in the near future should concentrate on the establishment of normative values or reference ranges for these parameters. In addition, studies should aim at a more local sampling protocol (e.g., abomasal biopsies or abomasal venous blood samples). Examining correlations between tissue, local venous, and serum concentrations might result in further insights in the pathogenesis of AD.

## Figures and Tables

**Table 1:** Quality control data for the ELISA test kits used in this study.

Test Kit	Detection Range	Precision (Coefficient of Variance [CV])	
		Intra-Assay	Inter-Assay
SP (CEA393Bo)	12.35–1000 pg/mL	CV < 10%	CV < 12%
VIP (CEA380Bo)	6.17–500 pg/mL	CV < 10%	CV < 12%
IL-1β (SEA563Bo)	15.6–1000 pg/mL	CV < 10%	CV < 12%

SP: substance P; VIP: vasoactive intestinal polypeptide.
